# The complete mitochondrial genome of the sand bubbler crab *Scopimera longidactyla* Shen 1932 (Decapoda: Dotillidae) and its phylogenetic analysis

**DOI:** 10.1080/23802359.2024.2346603

**Published:** 2024-05-02

**Authors:** Jaeyong Bae, Dalyoung Kim, Seongryul Lim, Sungkon Kim, Jung Soo Heo, Keun-Yong Kim, Biet Thanh Tran, Seongmin Kim

**Affiliations:** a Gyeonggi Province Maritime & Fisheries Research Institute, Ansan, Gyeonggi-do, Republic of Korea; b Department of Genetic Analysis, AquaGenTech Co., Ltd., Busan, Republic of Korea

**Keywords:** Mitochondrial DNA, genome sequencing, decapod crustaceans, molecular markers

## Abstract

The sand bubbler crab, *Scopimera longidactyla* Shen, 1932 (Arthropoda: Malacostraca: Decapoda: Thoracotremata: Dotillidae), is commonly found along tropical and subtropical sandy shores of China, Korea, and Taiwan. Ecologically, it plays an important role in the productivity of sandy shores through their feeding and burrowing activities. In this study, the first complete mitochondrial genome (mitogenome) of *S. longidactyla* was analyzed using next-generation sequencer. Its mitogenome, circular in structure, spans 15,965 bp with a GC content of 29.97%, consisting of 13 protein-coding genes, two ribosomal RNA genes, 22 transfer RNA genes, and one putative control region. Its mitogenome arrangement and composition are identical to its two congeners, *S. globosa* and *S. intermedia*. Phylogenetic analysis fully supports for the monophyly of the genus *Scopimera* and the sister relationship between *S. longidactyla* and *S. globosa*. The complete mitogenome of *S. longidactyla* and its phylogenetic implications will provide valuable insights for further studies in phylogenetic and evolutionary biology.

## 1. Introduction

A sand bubbler crab, *Scopimera longidactyla* Shen, 1932 (Arthropoda: Malacostraca: Decapoda: Thoracotremata: Dotillidae), is a member of small crustacean Brachyura (true crabs) of the genera *Scopimera*, commonly found along tropical and subtropical sandy shores, including the western coast of Korea (Kim 1973; Jang and Kim 1989; Wong et al. 2011). This brachyuran crab inhabits burrows in the upper tidal zone and emerges onto the surface to consume detritus and plankton in the lower tidal zone (Koga 1995). It plays an ecologically significant role as a deposit feeder and bioturbator to the marine ecosystem’s food chain (Wong et al. 2011; Ko and Lee 2012). Presently, only two mitochondrial genome (mitogenome) sequences of the genus *Scopimera* are available in the GenBank database (National Center for Biotechnology Information 1988). This study aimed to sequence, annotate, and characterize the complete mitogenome of *S. longidactyla* and explore its phylogenetic position within the family Dotillidae, which is expected to significantly contribute to research on the phylogenetic position of the genus *Scopimera*.

## 2. Materials and methods

**Figure 1.**
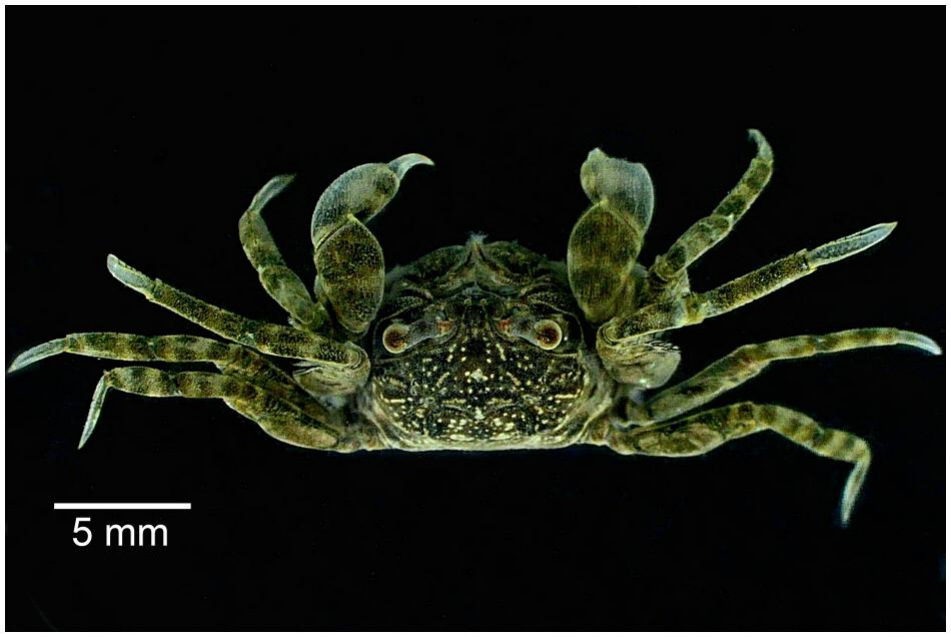
Specimen image of the sand bubbler crab, *Scopimera longidactyla*, collected from Daebu Island on the northwestern Coast of Korea. This photograph was taken by Dalyoung Kim in September 2022.

The morphology of *S. longidactyla* is characterized by a subglobose carapace with a strongly granular surface. Its ambulatory legs feature tympana on propodi, with the second leg standing out as the longest (Ko and Lee 2012). A specimen of adult *S. longidactyla* was collected from Daebu Island at the northwestern coast of Korea (37°14′28.14″N, 126°34′41.94″E) and deposited in the Marine Bioresource Collection of Gyeonggi Province Maritime & Fisheries Research Institute, Ansan, Republic of Korea (https://fish.gg.go.kr/) under a voucher number, GMFRI-C0008 (Figure 1).

Total genomic DNA of *S. longidactyla* was extracted from the muscle tissue of leg using the phenol-chloroform method with a lysis buffer (10 mM Tris-HCl, pH 8.0; 125 mM NaCl; 10 mM EDTA, pH 8.0; 1% SDS; 8 M Urea) according to Asahida et al. (1996). The isolated DNA underwent shearing using a S220 Ultra sonicator (Covaris, Woburn, MA, USA) and subsequent library preparation with the MGIEasy DNA library prep kit (MGI Tech Co., Ltd., Shenzhen, China). The resulting library was sequenced on an MGISEQ-2000 system (MGI Tech Co., Ltd.) with 150-bp paired-end runs (2 × 150).

A total of 69,061,136 raw reads underwent filtration to remove adapters, low-quality bases (Phred scores *Q* < 20), and short reads (< 25 bp) using cutadapt v. 1.9 (Martin 2011). The clean reads were subjected to *de novo* assembly using CLC Genomics Workbench v. 20.0.4 (CLC Bio, Aarhus, Denmark) with the following parameters: similarity fraction = 0.8; length fraction = 0.5. The assembled contigs with lengths greater than 10 kbp underwent BLASTN search (E-value = 0.001) implemented in the CLC Genomics Workbench against the nucleotide database at NCBI. The assembled contigs, displaying hits to mitochondrial genes and circularity (overlap at the beginning and end of the sequence), were extracted and validated by sequence alignment with the known mitogenome of *S. globosus* (GenBank accession number, LC535358) using the MAFFT alignment tool of Geneious v. 2021.1.1 (Biomatters, Inc., Auckland, New Zealand) at default parameters. The alignment output identified a single representative sequence with the highest similarity to the *S. globosus* mitogenome as a draft mitogenome sequence. To rectify any conflicts arising during assembly, the draft mitogenome was used as a reference to map the clean reads using the "Map to Reference" option with default settings in Geneious v. 2021.1.1. A total of 93,300 reads were mapped to the reference mitogenome with the mean coverage depth of 867.8× (Supplementary Figure S1).

The consensus sequence, based on the highest quality threshold of the base call for each nucleotide position, was annotated using the MITOS web server (Bernt et al. 2013). Transfer RNA (tRNA) genes were identified by tRNAscan-SE 1.21 (Lowe and Eddy 1997). The order and orientation of the genes were drawn using GenomeVx (Conant and Wolfe 2008). The annotated sequence of the complete mitogenome of *S. longidactyla* was deposited in GenBank under the accession number, OR872329.

To determine the phylogenetic position of *S. longidactyla*, eight available mitogenome sequences of the family Dotillidae and two outgroup species of the family Grapsidae, *Pachygrapsus crassipes* (KC878511) and *Grapsus tenuicrustatus* (KT878721), were obtained from GenBank database (Table 1). These mitogenome sequences were aligned using ClustalW multiple alignment function of Bioedit v.7.2.5 (Hall 1999) with default parameters. Phylogenetic analysis was conducted based on concatenated nucleotide sequence alignment of 13 protein-coding genes (PCGs) from these aligned sequences. The GTRGAMMAI model, a general time-reverse model incorporating invariant sites and gamma distribution, was selected as the optimal phylogenetic model by JModelTest v. 2 (Darriba et al. 2012). This model was employed for constructing the maximum likelihood (ML) tree and executing the Bayesian inference (BI) analysis. The ML analysis was performed with RAxML 7.0.4 using 1000 nonparametric bootstrap inferences (Stamatakis 2006; Stamatakis et al. 2008). For BI analysis, MrBayes v. 3.1.2 was utilized, employing four independent Markov chains with 1,000,000 generations and discarding the initial 25% as burn-in (Ronquist et al. 2012). Stationarity was reached when the average standard deviation of split frequencies was below 0.01 (ASDSF = 0.001480). The resulting tree was visualized using TreeViewX v. 0.5.0 (Page 2002).

**Table 1.** List of accession number and publication used in the phylogenetic tree.

Family	Species	GenBank accession number	References
Dotillidae	*Dotilla wichmanni*	MH183129	Chen et al. (2018)
	*Ilyoplax deschampsi*	JF909979	Ji et al. (2014)
	*Ilyoplax integra*	LC715474	Kobayashi et al. (2023)
	*Ilyoplax pusilla*	LC715475	Kobayashi et al. (2023)
	*Scopimera globosa*	LC535358	Kobayashi et al. (2021)
	*Scopimera intermedia*	MW165226	Wang et al. (2021)
	*Scopimera longidactyla*	OR872329	Present study
	*Tmethypocoelis choreutes*	LC715478	Kobayashi et al. (2023)
Grapsidae (Outgroup)	*Grapsus tenuicrustatus*	KT878721	Sung et al. (2016)
	*Pachygrapsus crassipes*	KC878511	Yu et al. (2014)

## 3. Results

**Figure 2.**
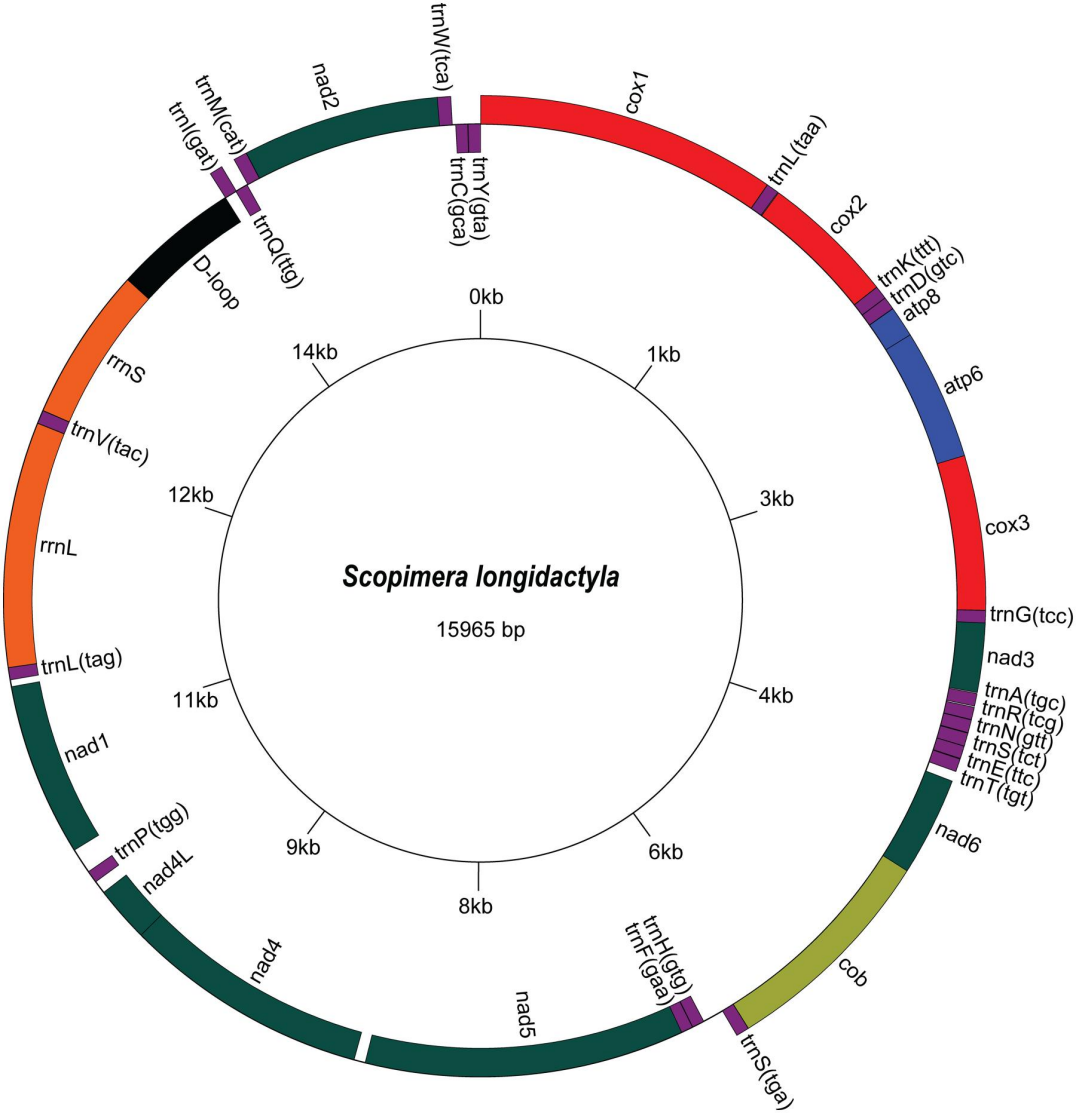
The circular mitogenome map of *Scopimera longidactyla* (GenBank Accession No. OR872329). Genes encoded on the reverse strand and forward strand are illustrated inside the circle and outside the circle, respectively.

The complete mitogenome of *S. longidactyla* was 15,965 bp in length, circular, and double-stranded, comprising 13 PCGs, two ribosomal RNA genes, 22 transfer RNA genes, and one putative control region (Figure 2). More than half of these genes were encoded on the heavy (H) strand with nine PCGs and 14 tRNAs. All PCGs initiated with ATN codons, except for *nad2*, which starts with GTG. They terminated with either TAA or incomplete stop codons T (in *cox1*, *cox2*, *cox3*, *cob*, *nad2*, *nad5*). The overall base composition was A (34.74%), T (35.29%), G (11.99%), and C (17.98%), with a total proportion of G + C of 29.97%.

**Figure 3.**
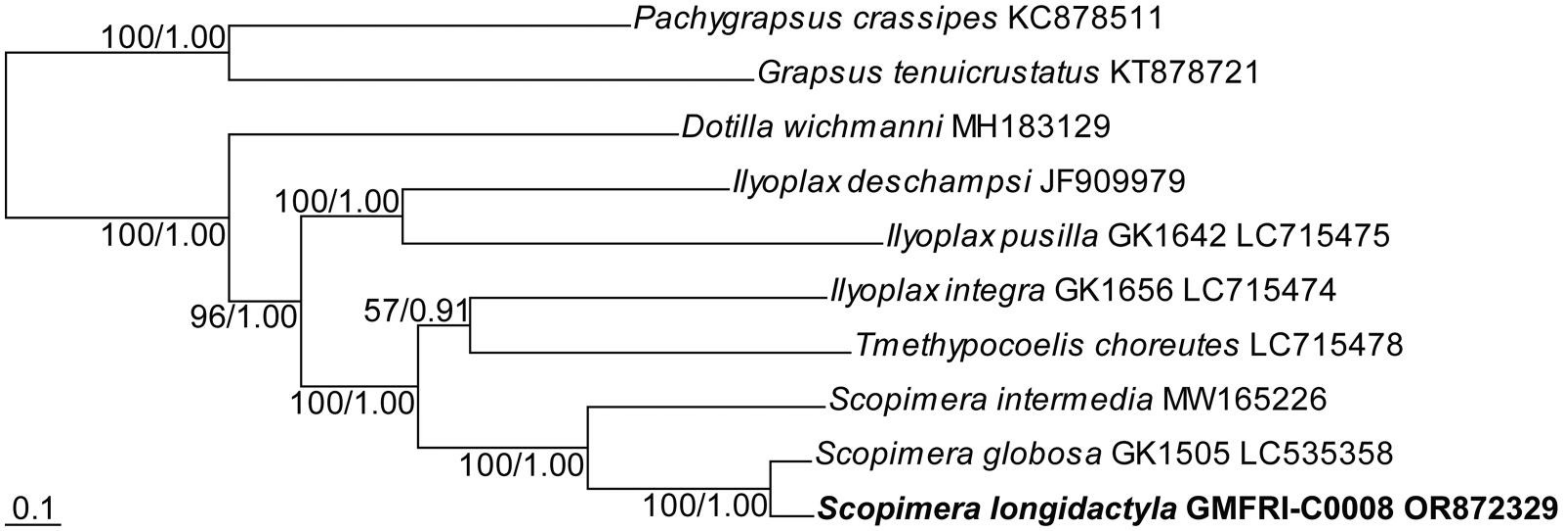
Maximum likelihood (ML) tree based on concatenated nucleotide sequences from all protein-coding genes of the complete mitogenomes of the family Dotillidae. Numeric values at nodes are ML bootstraps followed by the Bayesian posterior probabilities. The newly assembled mitogenome of the sand bubbler crab, *Scopimera longidactyla* was highlighted in bold. Two species, *Pachygrapsus crassipes* and *Grapsus tenuicrustatus*, belonging to the family Grapsidae, were selected as the outgroup. Scale bar represents nucleotide substitutions per site.

The ML tree was shown in Figure 3. Its topology was identical to that of the phylogenetic tree derived from BI analysis. The phylogenetic analysis fully supported the monophyly of the genus *Scopimera* and the sister relationship between *S. longidactyla* and *S. globosa* (BS = 100% and posterior probability, PP = 1).

## 4. Discussion and conclusion

The high-throughput sequencing analysis reveals the first complete mitogenome of *S. longidactyla*, spanning 15,965 bp in length. Its mitochondrial gene composition aligns with typical crustacean Brachyura, comprising 13 PCGs, 22 tRNAs, and two rRNAs (Kobayashi et al. 2021; Wang et al. 2021; Zhang et al. 2023). In addition, its mitogenome arrangement and composition are identical to its two congeners, *S. globosa* and *S. intermedia* (Kobayashi et al. 2023). Although the gene order between *trnE* and *nad1* of three species in the genus *Scopimera* particularly differs with three dotillid species (*Dotilla wichmanni*, *Ilyoplax pusilla*, and *I. deschampsi*), this gene pattern is not unique to *Scopimera*, as it is also shared with two other species, *Tmethypocoelis choreutes* and *I. integra*, within the family Dotillidae. The phylogenetic analysis provides full support for the monophyly of the genus *Scopimera*, as well as the sister relationship observed between *S. longidactyla* and *S. globosa*. These results will contribute to future studies on the evolutionary and phylogenetic analysis including *S. longidactyla.*

## Supplementary Material

Supplemental Material

## Data Availability

The data that support the findings of this study are openly available in GenBank of NCBI under the accession number OR872329. Raw reads have been deposited under NCBI BioProject (PRJNA1041591), BioSample (SAMN38288372), and SRA (SRR26857270).
