# Supplementary material for: The complete mitochondrial genome of the sand bubbler crab Scopimera longidactyla Shen 1932 (Decapoda: Dotillidae) and its phylogenetic analysis
Source: Mitochondrial DNA B Resour. 2024 May 2;9(5):574–7. doi: 10.1080/23802359.2024.2346603 (PMC11067553; doi:10.1080/23802359.2024.2346603)
Supplement: Supplemental Material [file TMDN_A_2346603_SM1624.pdf]

Supplementary materials

The complete mitochondrial genome of the sand bubbler crab *Scopimera longidactyla* Shen 1932 (Decapoda: Dotillidae) and its phylogenetic analysis

Jaeyong Bae[a], Dalyoung Kim[a], Seongryul Lim[a], Sungkon Kim[a], Jung Soo Heo[b], Keun-Yong Kim[b], Biet Thanh Tran[b*], and Seongmin Kim[a,*]

[a]Gyeonggi Province Maritime & Fisheries Research Institute, Ansan, Gyeonggi-do 15651, Republic of Korea; JYB (baejy318@gg.go.kr, ORCID no. 0009-0006-3527-3975); DYK (aqualife@gg.go.kr, 0009-0007-7337-3798); SRL (cafrio@gg.go.kr, 0000-0001-6899-3457); SKK (sung526@gg.go.kr, 0009-0003-0951-725X); SMK (smkim1020@gg.go.kr, 0009-0008-4930-4263)

[b]Department of Genetic Analysis, AquaGenTech Co., Ltd., Busan 48228, Republic of Korea; JSH (dgyjs2@daum.net, 0000-0001-9467-4900); KYK (koby0323@hanmail.net, 0000-0002-7647-3766); BTT (tranthanhbiet2502@gmail.com, 0000-0002-1849-6089)

*Co-corresponding author:

Seongmin Kim

Tel: +82-10-8801-0787; Fax: +82-31-890-4160; E-mail: smkim1020@gg.go.kr

Address: 71, Gaegeonneo-gil, Danwon-gu, Ansan, Gyeonggi-do 15651, Republic of Korea

Biet Thanh Tran

Tel: +82-10-4612-4278; Fax: +82-51-623-0307; E-mail: tranthanhbiet2502@gmail.com

Address: Suyeong-dong, Suyeong-gu, Busan 48228, Republic of Korea

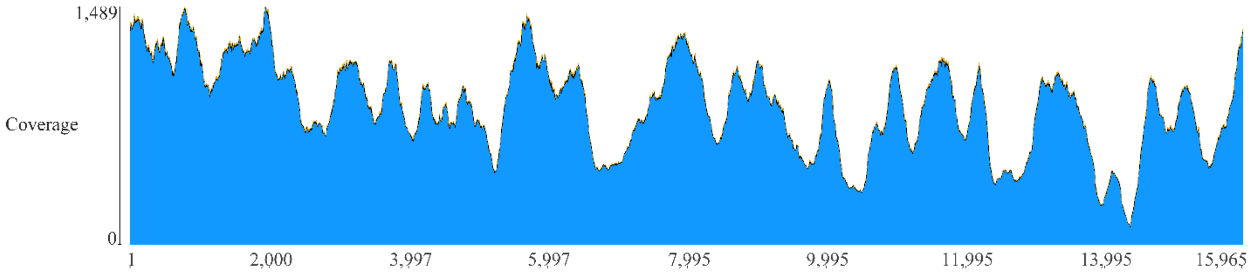

Figure S1. Coverage depth plot of the assembled mitochondrial genome of *Scopimera longidactyla* (GenBank accession no. OR872329). The horizontal axis represents the nucleotide position, and the vertical axis represents the read mapping depth.
